# Delay-dependent stability and stabilization criteria for T–S fuzzy singular systems with interval time-varying delay by improved delay partitioning approach

**DOI:** 10.1186/s40064-016-1982-2

**Published:** 2016-03-22

**Authors:** Chao Sun, Fuli Wang, Xiqin He

**Affiliations:** College of Information Science and Engineering, Northeastern University, Shenyang, 110819 China; School of Science, University of Science and Technology Liaoning, Anshan, 114051 China

**Keywords:** Fuzzy singular system, Delay-partitioning approach, Interval time-varying delay, Linear matrix inequalities (LMIs)

## Abstract

This paper deals with the stability analysis and fuzzy stabilizing controller design for a class of Takagi–Sugeno fuzzy singular systems with interval time-varying delay and linear fractional uncertainties. By decomposing the delay interval into two unequal subintervals and seeking a appropriate *ρ*, a new Lyapunov–Krasovskii functional is constructed to develop the improved delay-dependent stability criteria, which ensures the considered system to be regular, impulse-free and stable. Furthermore, the desired fuzzy controller gains are also presented by solving a set of strict linear matrix inequalities. Compared with some existing results, the obtained ones give the result with less conservatism. Finally, some examples are given to show the improvement and the effectiveness of the proposed method.

## Background

During the past few decades, fuzzy technique has been widely used in nonlinear system modelling, especially for systems with incomplete plant information. Thus, a number of significant results have been reported to solve the different problems of fuzzy systems, such as stability analysis (Chadli et al. 2014; Su et al. 2013, 2014b), filter design (Shi et al. 2015; Su et al. 2014a, 2015), robust control (Mourad et al. 2013; Tian et al. 2010), etc. Among these conclusions, it is worth mentioning that, the problem of Hankel-norm output feedback controller design for T–S fuzzy stochastic systems have been investigated in Su et al. (2014b). For a class of T–S fuzzy switched systems with stochastic perturbation, the dissipativity-based filtering problem was considered in Shi et al. (2015). In addition, the fault detection filtering problem have been solved for nonlinear switched stochastic system in the T–S fuzzy framework in Su et al. (2015). Recently, a wider class of fuzzy systems that are described by the singular form have been studied, where the model is the extended of T–S fuzzy systems (Taniguchi et al. 2000). It is known that a singular model can describes a practical system better than a standard dynamic model. So fuzzy singular model provides a new way to the analysis and synthesis of the nonlinear singular system and can be found in many applications, because it can combine the flexibility of fuzzy logic theory and fruitful linear singular system theory into a unified framework to approximate complex nonlinear singular systems, for details see Fridman (2002) and Lin et al. (2006). Meanwhile, time delays always exist in many dynamical systems and delays are the sources of poor stability and deteriorated performance of a system. Therefore, lots of stability analysis and control synthesis results (Wang et al. 2014; Mourad et al. 2013; Huang 2013; Han et al. 2012; Zhang et al. 2009) have been reported for T–S fuzzy singular systems with time-delay. It should be pointed out that almost all of the existing results on fuzzy systems with time delays, the maximum allowable delay bound has been used as an important performance index for measuring the conservatism of the obtained conditions.

On the other hand, in order to reduce the conservativeness of the delay-dependent criteria for fuzzy systems, input–ouput approach (Su et al. 2013; Zhao et al. 2013), delay partitioning method (Yang et al. 2015; Xia et al. 2014), convex combination technique (Su et al. 2014a; An and Wen 2011; Peng and Fei 2013; Park et al. 2011), and free weighting matrices approach (Souza et al. 2014; Liu et al. 2010; Tian et al. 2010) have been well used. The most noteworthy is the delay partitioning approach: the delay interval is divided into multiple uniform or non-uniform segments. It has been proved that less conservative results may be expected with the increasing of delay-partitioning segments. Recently, by non-uniformly dividing the time delay into multiple segments, An and Wen (2011) has established less conservative delay-dependent stability criteria than those in Li et al. (2009) using a convex way for uncertain T–S fuzzy systems with interval time-varying delay. Based on the input–output technique and delay partitioning approach, some new stability conditions of discrete-time T–S fuzzy systems with time delays have been proposed by applying scaled small-gain theorem in Su et al. (2013) , while an induced $\ell _{2}$ performance is guaranteed. On the basis of delay-partitioning approach and new integral inequality established by reciprocally convex approach in Park et al. (2011) and Peng and Fei (2013) has developed less conservative stability criteria than those in Peng et al. (2011), Lien et al. (2007) and Tian and Chen (2006) for uncertain T–S fuzzy delay system. However, an important characteristic of fuzzy singular systems is the possible impulse behavior, which is harmful to the physical system and is undesired in system control. It’s means that the aforementioned delay partitioning approach and the obtained results can not be directly applied to fuzzy singular system with additional algebraic constraints, because it requires considering not only stability, but also regularity and impulse-free conditions. Therefore, the motivation of this study is mainly focus on how to improve the delay partitioning approach and reduce the conservativeness of existing results for fuzzy singular systems because of its theoretical and practical significance.

More recently, some research works on stability analysis (Mourad et al. 2013; Zhang et al. 2009; Chadli et al. 2014; Wang et al. 2014) and controller design (Zhu et al. 2016; Ma et al. 2015; Zhao et al. 2015; Han et al. 2012) have been extended for T–S fuzzy singular systems with time-varying delay. In Zhang et al. (2009), the problems of delay-dependent stability and $H_{\infty }$ control were discussed utilizing model transformation techniques, but model transformation may lead to considerable conservativeness. In Han et al. (2012), the problems of sliding mode control for fuzzy descriptor systems were presented using delay partitioning approach, but the time-delay is constant. Using free-weight matrix method, Mourad et al. (2013) discussed the problems of delay-dependent stability and $L_{2}-L_{\infty }$ control, however, the free-weighting matrices may be redundant and increase the computational burden in case of stability analysis for deterministic delay systems. In Chadli et al. (2014), by using quadratic method, sufficient conditions on stability and stabilization were proposed in terms of LMI for uncertain T–S fuzzy singular systems. Based on delay partitioning approach, some less conservative stability and stabilization criteria for fuzzy singular systems with time-varying delay have been investigated in Wang et al. (2014). In Ma et al. (2015), a delay-central-point method was presented to develop less conservative delay-dependent conditions for memory dissipative control for fuzzy singular time-varying delay systems under actuator saturation.

It is well-known that the challenges of deriving a less conservative result are to construct an appropriate LKF that includes more useful state information and to reduce the enlargement in bounding the derivative of LKF as much as possible. Inspired by the methods mentioned above, when revisiting the stability problem for T–S fuzzy singular systems with interval time-varying delay, we find that the existing works still leave plenty of room for improvement on the reduction of conservatism for the following reasons. (1) All the given stability conditions in Han et al. (2012), Mourad et al. (2013), and Wang et al. (2014) are not all in strict LMIs form due to equality constraints, which cannot be solved directly using standard LMI procedures; (2) In Wang et al. (2014), the integral item $-\int _{t-\tau _{2}}^{t-\tau _{1}}{\dot{x}}^{T}(s)E^{T}RE{\dot{x}}(s)ds$ is directly magnified as $-(\tau _{2}-\tau (t))\int _{t-\tau _{2}}^{t-\tau (t)}{\dot{x}}^{T}(s)E^{T}RE{\dot{x}}(s)ds-(\tau (t)-\tau _{1})\int _{t-\tau (t)}^{t-\tau _{2}}{\dot{x}}^{T}(s)E^{T}RE{\dot{x}}(s)ds$, some useful time-varying delay-dependent integral items are ignored in the derivation of results; (3) More free-weighting matrices are employed to deduce the stabilization results in Yang et al. (2015) and Mourad et al. (2013), which have not considered the gain variations might be caused by the inaccuracies of controller implementation. The objective of this paper is to revisit the delay-dependent stability analysis and give new stabilization criteria by improved delay partitioning approach.

The main contributions of this paper lie in that, firstly, by seeking an appropriate $\rho$, a maximum admissible upper bound of the time delay can be obtained for T–S fuzzy singular systems with interval time-varying delay. The tunable parameter $\rho$ which divide $[\tau _{1},\tau _{2}]$ into two variable subintervals plays a crucial role in reducing the conservativeness of stability conditions. Secondly, new LKF is established by partitioning time delay $[0,\tau _{1}]$ into *N* segments, and the time-varying delay $x(t-\frac{n}{N}\tau _{1})$ is included in the LKF, which takes fully account of the relationship between the state vectors $x(t-\frac{n}{N}\tau _{1})$ and $x(t-\tau _{\rho })$. Thirdly, some new results on tighter bounding inequalities have been employed to reduce the enlargement in bounding the derivative of LKF when designing the controller with linear fractional uncertainties. Then, the newly developed conditions of stability and stabilization are expected to be less conservative than the previous ones.

The rest of this paper is organized as follows. The system description and some useful lemmas are presented in “Problem formulation” section. In “Main results” section, we show the results on stability conditions and fuzzy controller design scheme. In “Numerical examples” section, several numerical examples are given to demonstrate the effectiveness and merits of the proposed methods. Finally, a brief conclusion is drawn in “Conclusion”.

Notations: Throughout the paper, ${\mathbb{R}}^{n}$ denotes the n-dimensional real Euclidean space; *I* denotes the identity matrix; the superscripts *T* and $-1$ stand for the matrix transpose and inverse, respectively; notation $X>0(X\ge 0)$ means that matrix *X* is real symmetric positive definite (positive semi-definite); $\Vert \cdot \Vert$ is the spectral norm. If not explicitly stated, all matrices are assumed to have compatible dimensions for algebraic operations. The symbol “$*$” stands for matrix block induced by symmetry.

## Problem formulation

Consider a class of nonlinear singular system with interval time-varying delay, which can be represented by the following extended T–S fuzzy singular model:

1 $$\begin{aligned} \left\{ \begin{aligned} &E{\dot{x}}(t)=\sum _{i=1}^{r}\mu _{i}(\xi (t))\{ (A_{i}+\Delta A_{i}(t))x(t)+(A_{\tau i}+\Delta A_{\tau i}(t))x(t-\tau (t))+B_{i}u(t)\} \\ &x(t)=\sum _{i=1}^{r}\mu _{i}(\xi (t))\phi _{i}(t), \quad \forall t\in [-\tau _{2},0]. \end{aligned}\right. \end{aligned}$$

where $x(t)\in {\mathbb{R}}^{n}$ is the state vector, $u(t)\in {\mathbb{R}}^{m}$ is the control input vector. The fuzzy basis functions are given by $\mu _{i}(\xi (t))=\beta _{i}(\xi (t))/\sum _{j=1}^{r}\beta _{j}(\xi (t))$ with $\beta _{i}(\xi (t))= \prod _{i=1}^{p}M_{ij}(\xi (t))$, where $M_{ij}$ is fuzzy sets, $M_{ij}(\xi _{j}(t))$ represents the grade of membership of $\xi _{j}(t)$ in $M_{ij}$. Here, it is easy to find that $\beta _{i}(\xi (t))\ge 0, (i=1,2,\ldots ,r)$, $\sum _{j=1}^{r}\beta _{j}(\xi (t))>0$ and $\mu _{i}(\xi (t))\ge 0, (i=1,2,\ldots ,r)$, $\sum _{j=1}^{r}\mu _{j}(\xi (t))=1$ for all $t>0$, *r* is the number of IF–THEN rules. $\xi _{1}(t),\ldots ,\xi _{p}(t)$ are the premise variables, which do not depend on the input variable *u*(*t*). $\phi _{i}(t)$ is a vector-valued initial continuous function defined on the interval $[-\tau _{2},0]$. $E\in {\mathbb{R}}^{n\times n}$ is a constant matrix, which may be singular, that is, rank$(E)=g\le n$. $A_{i}$, $A_{\tau i}$, $B_{i}$ are the constant real matrices of appropriate dimensions. $\Delta A_{i}(t)$ and $\Delta A_{\tau i}(t)$ denote the norm-bounded parameter uncertainties in the system and are defined as:

2 $$\begin{aligned}{}[\Delta A_{i}(t) \ \Delta A_{\tau i}(t)]=M_{i}F(t)[N_{1i} \ N_{2i}] \end{aligned}$$

where $M_{i}$, $N_{1i}$ and $N_{2i}$ are known matrices, *F*(*t*) is unknown time-varying matrix, which satisfies $F^{T}(t)F(t)\le I$. The delay $\tau (t)$ in above systems is assumed to be interval time varying and satisfies

3 $$\begin{aligned} \tau _{1} \le \tau (t)\le \tau _{2}, \quad \dot{\tau }(t)\le d \end{aligned}$$

where $\tau _{1}$, $\tau _{2}$ and *d* are constants.

Before proceeding further, we will introduce some definitions and lemmas to be needed in the development of main results throughout this paper. Consider an unforced singular time-delay system described by

4 $$\begin{aligned} \left\{ \begin{aligned} &E{\dot{x}}(t)= Ax(t)+A_{\tau }x(t-\tau (t)) \\ &x(t)= \phi (t), t\in [-\tau _{2},0] \\ \end{aligned} \right. \end{aligned}$$

### **Definition 1**

(Xu et al. 2002)

1. The pair (*E*, *A*) is said to be regular if $\det (sE-A)$ is not identically zero.
2. The pair (*E*, *A*) is said to be impulse free if $deg (\det (sE-A))= rank(E)$.
3. The pair (*E*, *A*) is said to be asymptotically stable, if all roots of $\det (sE-A)=0$ lie inside the unit disk with center at the origin.
4. The delayed singular system (4) is said to be admissible if the pair (*E*, *A*) is regular, impulse free and asymptotically stable.

### **Definition 2**

(Xu et al. 2002)

1. The singular system (4) is said to be regular and impulse free if the pair (*E*, *A*) is regular and impulse free.
2. The singular system (4) is said to be asymptotically stable, if for any $\varepsilon >0$, there exists a scalar $\delta (\varepsilon )>0$ such that for any compatible initial conditions, $\phi (t)$ with $sup_{-\tau (t)\le t\le 0}\Vert \phi (t)\Vert <\delta (\varepsilon )$, the solution *x*(*t*) of (4) satisfies $\Vert x(t)\Vert <\varepsilon$ for $t\ge 0$ and $\lim _{t\rightarrow \infty }x(t)=0$.

### **Lemma 3**

(Liu 2013) *For any positive semi-definite matrices*$X=(X_{ij})_{3 \times 3}\ge 0$, *the following integral inequality holds:*

$$\begin{aligned} -\int _{t-\tau (t)}^{t}&{\dot{x}}^{T}(s)X_{33}{\dot{x}}(s)ds \le \int _{t-\tau (t)}^{t} \beta (t,s) \begin{bmatrix} X_{11}&\quad X_{12}&\quad X_{13} \\ X_{12}^{T}&\quad X_{22}&\quad X_{23} \\ X_{13}^{T}&\quad X_{23}^{T}&\quad 0 \end{bmatrix}\beta ^{T}(t,s)ds \end{aligned}$$

*where*$$\beta (t,s)=[x^{T}(t)\ x^{T}(t-\tau (t)) \ {\dot{x}}^{T}(s) ]$$.

### **Lemma 4**

(Han 2005) *For any constant matrix*$X\in {\mathbb{R}}^{n\times n}$, $X=X^{T}>0$, *scalar*$r>0$, *and vector function*${\dot{x}}:[-r,0]\rightarrow {\mathbb{R}}^{n}$*such that the following integration is well defined, then*

$$\begin{aligned} -r\int _{-r}^{0}{\dot{x}}^{T}(t+s)&X{\dot{x}}(t+s)ds \le [x^{T}(t)\ x^{T}(t-r)] \begin{bmatrix} -X&\quad X \\ X&\quad -X \end{bmatrix} \begin{bmatrix} x(t) \\ x(t-r) \end{bmatrix} \end{aligned}$$

### **Lemma 5**

(Xie 1996) *Given a symmetric matrix*$\Omega$*and matrices*$\Gamma$, $\Xi$*with appropriate dimensions,*$\Omega +\Gamma \Delta \Xi +\Xi ^{T}\Delta ^{T}\Gamma ^{T}<0$*for all*$\Delta$*satisfying*$\Delta ^{T}\Delta \le I$, *if and only if there exists a scalar*$\varepsilon >0$*such that*$\Omega +\varepsilon \Gamma \Gamma ^{T}+\varepsilon ^{-1}\Xi ^{T}\Xi <0$

### **Lemma 6**

(Fridman 2002) *If a functional*$V:C_{n}[-\tau ,0]\rightarrow {\mathbb{R}}$*is continuous and*$x(t,\phi )$*is a solution to* (4), *we define*$\dot{V}(\phi )=\lim \nolimits _{h\rightarrow 0^{+}}sup \frac{1}{h}(V(x(t+h,\phi ))-V(\phi ))$. *Denote the system parameters of* (4) *as*

$$\begin{aligned} (E,\ A, \ A_{\tau })=\Bigg (\begin{bmatrix} I_{g}&\quad 0 \\ 0&\quad 0 \end{bmatrix}, \begin{bmatrix} A_{11}&\quad A_{12} \\ A_{21}&\quad A_{22} \end{bmatrix}, \begin{bmatrix} A_{\tau 11}&\quad A_{\tau 12} \\ A_{\tau 21}&\quad A_{\tau 22} \end{bmatrix}\Bigg ) \end{aligned}$$

*Assume that the singular system* (4) *is regular and impulse free,*$A_{22}$*is invertible,*$\rho (A_{22}^{-1}A_{\tau 22})<1$. *Then, the system* (4) *is stable if there exists positive numbers*$\alpha , \mu , \nu$*and a continuous function,*$V:C_{n}[-\tau ,0]\rightarrow {\mathbb{R}}$, *such that*

$$\begin{aligned} \mu \Vert \phi _{1}(0)\Vert ^{2}\le V(\phi )\le \nu \Vert \phi \Vert ^{2}, \dot{V}(x_{t})\le -\alpha \Vert x_{t}\Vert ^{2} \end{aligned}$$

*where*$x_{t}=x(t+\theta )$*with*$\theta \in [-\tau , 0]$*and*$\phi =[\phi _{1}^{T} \ \phi _{2}^{T}]$*with*$\phi _{1}\in {\mathbb{R}}^{q}$.

## Main results

### Delay-dependent admissibility

In this section, we suggest to develop a delay-dependent stability condition for the nominal unforced fuzzy singular system of (1), which can be written as

5 $$\begin{aligned} \left\{ \begin{aligned} &E{\dot{x}}(t)= A(t)x(t)+A_{\tau }(t)x(t-\tau (t)) \\ &x(t)= \phi (t), t\in [-\tau _{2},0] \end{aligned}\right. \end{aligned}$$

where $A(t)=\sum _{i=1}^{r}\mu _{i}(\xi (t))A_{i}$, $A_{\tau }(t)=\sum _{i=1}^{r}\mu _{i}(\xi (t))A_{\tau i}$. In order to derive a maximum admissible upper bound of system (5), the delay interval $[\tau _{1},\tau _{2}]$ is divided into two subintervals with unequal width as Case I: $[\tau _{1},\tau _{\rho }]$ and Case II: $[\tau _{\rho },\tau _{2}]$, where $\tau _{\rho }=\tau _{1}+\rho \delta$, $\delta =\tau _{2}-\tau _{1}$, $0<\rho <1$. Based on the Lyapunov–Krasovskii stability theorem, the following result is obtained.

#### **Theorem 7**

*For the given scalars*$\tau _{1}$, $\tau _{2}$, *dand tuning parameter*$\rho$, *system* (5) *is admissible for any time-varying delay*$\tau (t)$*satisfying* (3), *if there exist matrices*$P_{1}>0$, $Q_{n}>0$, $W_{n}>0\; (n=1,2,\ldots ,N)$, $\Lambda ^{T}(Y_{ij})_{3\times 3}\Lambda =\hat{Y}\ge 0$, $\Lambda ^{T}(Z_{ij})_{3\times 3}\Lambda =\hat{Z}\ge 0$, $\Lambda ={\mathrm{diag}}\{E,E,E\}$, $S_{1}>0$, $S_{2}>0$, $S_{3}>0$, $R_{1}>0$, $R_{2}>0$, *some appropriate dimension matricesS*, $P_{2}$, $P_{3}$*and the constant matrixRsatisfying*$E^{T}R=0$*such that the following set of LMIs hold:*

6 $$\begin{aligned} \Theta ^{i}=\begin{bmatrix} \Theta _{11}^{i}&\quad \Theta _{12}^{i}\\ *&\quad \Theta _{22}^{i} \end{bmatrix}<0 \end{aligned}$$

*and*

7 $$\begin{aligned} R_{1}-Y_{33}\ge 0, \quad R_{2}-Z_{33}\ge 0 \end{aligned}$$

*where*

8 $$\begin{aligned} \Theta _{11}^{i}&=\begin{bmatrix} \Theta _{1,1}^{i}&\quad E^{T}W_{1}E&\quad \cdots&\quad 0 \\ *&\quad \Theta _{2,2}&\quad \cdots&\quad 0 \\ \vdots&\quad \vdots&\quad \ddots&\quad \vdots \\ *&\quad *&\quad \cdots&\quad \Theta _{n,n} \end{bmatrix} \end{aligned}$$

9 $$\begin{aligned} \Theta _{12}^{i}&=\begin{bmatrix} 0&\quad 0&\quad \Theta _{(1,N3)}^{i}&\quad 0&\quad \Theta _{(1,N5)}^{i} \\ 0&\quad 0&\quad 0&\quad 0&\quad 0 \\ \vdots&\quad \vdots&\quad \vdots&\quad \vdots&\quad \vdots \\ E^{T}W_{N}E&\quad 0&\quad 0&\quad 0&\quad 0 \\ \end{bmatrix} \end{aligned}$$

10 $$\begin{aligned} \Theta _{22}^{i}&=\begin{bmatrix} \Theta _{(N1,1)}&\quad \Theta _{(N1,2)}&\quad \Theta _{(N1,3)}&\quad 0&\quad 0 \\ *&\quad \Theta _{(N2,2)}&\quad \Theta _{(N2,3)}&\quad \Theta _{(N2,4)}&\quad 0 \\ *&\quad *&\quad \Theta _{(N3,3)}&\quad \Theta _{(N3,4)}&\quad \Theta _{(N3,5)}^{i} \\ *&\quad *&\quad *&\quad \Theta _{(N4,4)}&\quad 0 \\ *&\quad *&\quad *&\quad *&\quad \Theta _{(N5,5)} \\ \end{bmatrix} \end{aligned}$$

*with*

11 $$\begin{aligned} \Theta _{1,1}^{i}&=P_{2}^{T}A_{i}+A_{i}^{T}P_{2}+Q_{1}+S_{1}-E^{T}W_{1}E \nonumber \\ \Theta _{n,n}&=-Q_{n-1}-E^{T}W_{n-1}E+Q_{n}-E^{T}W_{n}E \nonumber \\ \Theta _{(1,N3)}^{i}&=P_{2}^{T}A_{\tau i}, \Theta _{(1,N5)}^{i}=E^{T}P_{1}+SR^{T}-P_{2}^{T}+A_{i}^{T}P_{3} \nonumber \\ \Theta _{(N1,1)}&=-Q_{N}-E^{T}W_{N}E+S_{2}+\rho \delta \hat{Y}_{11}+\hat{Y}_{13}+\hat{Y}_{13}^{T} \nonumber \\ \Theta _{(N2,2)}&=S_{3}-S_{2}+ \rho \delta \hat{Y}_{22}-\hat{Y}_{23}-\hat{Y}_{23}^{T}+(1-\rho )\delta \hat{Z}_{11}+\hat{Z}_{13}+\hat{Z}_{13}^{T} \nonumber \\ \Theta _{(N3,5)}&=A_{\tau i}^{T}P_{3}, \Theta _{(N4,4)}=-S_{3}+ (1-\rho ) \delta \hat{Z}_{22}-\hat{Z}_{23}-\hat{Z}_{23}^{T} \nonumber \\ \Theta _{(N5,5)}&=\sum _{n=1}^{N}h^{2}W_{n}+\rho \delta R_{1}+(1-\rho )\delta R_{2}-P_{3}-P^{T}_{3} \end{aligned}$$

***Case I****when*$$\tau _{1} \le \tau (t) \le \tau _{\rho }$$

12 $$\begin{aligned} \Theta _{(N1,3)}&=\Theta ^{T}_{(N2,3)}=\rho \delta \hat{Y}_{12}-\hat{Y}_{13}+\hat{Y}_{23}^{T}, \Theta _{(N3,4)}=0 \nonumber \\ \Theta _{(N2,4)}&=(1-\rho )\delta \hat{Z}_{12}-\hat{Z}_{13}+\hat{Z}_{23}^{T}, \Theta _{(N1,2)}=0 \nonumber \\ \Theta _{(N3,3)}&=-(1-d)S_{1}+\rho \delta \hat{Y}_{11}+\hat{Y}_{13}+\hat{Y}_{13}^{T}+\rho \delta \hat{Y}_{22}-\hat{Y}_{23}-\hat{Y}_{23}^{T} \end{aligned}$$

***Case II****when*$\tau _{\rho } \le \tau (t) \le \tau _{2}$

13 $$\begin{aligned} \Theta _{(N1,2)}&=\rho \delta \hat{Y}_{12}-\hat{Y}_{13}+\hat{Y}_{23}^{T}, \Theta _{(N2,4)}=\Theta _{(N1,3)}=0\nonumber \\ \Theta _{(N2,3)}&=\Theta _{(N3,4)}=(1-\rho )\delta \hat{Z}_{12}-\hat{Z}_{13}+\hat{Z}_{23}^{T}\nonumber \\ \Theta _{(N3,3)}&=-(1-d)S_{1}+(1-\rho )\delta (\hat{Z}_{11}+\hat{Z}_{22})+\hat{Z}_{13}+\hat{Z}_{13}^{T} -\hat{Z}_{23}-\hat{Z}_{23}^{T} \end{aligned}$$

#### *Proof*

The proof of this theorem is divided into two parts. The first one is concerned with the regularity and the impulse free characterizations, and the second one treats the stability property of system (5). Since rank$(E)=g\le n$, there must exist two invertible matrices $G \in {\mathbb{R}}^{n\times n}$ and $H \in {\mathbb{R}}^{n\times n}$ such that

14 $$\begin{aligned} \tilde{E}=GEH=\begin{bmatrix} I_{g}&\quad 0 \\ 0&\quad 0 \end{bmatrix} \end{aligned}$$

Similar to (14), we define

15 $$\begin{aligned} \tilde{A}_{i}=GA_{i}H=\begin{bmatrix} \tilde{A}_{i11}&\quad \tilde{A}_{i12} \\ \tilde{A}_{i21}&\quad \tilde{A}_{i22} \end{bmatrix}, \quad {\tilde{P}}=G^{-T}P_{2}H=\begin{bmatrix} \tilde{P}_{11}&\quad \tilde{P}_{12} \\ \tilde{P}_{21}&\quad \tilde{P}_{22} \end{bmatrix} \end{aligned}$$

Since $\Theta ^{i}<0$ and $Q_{1}>0$, $S_{1}>0$, we can formulate the following inequality easily:

16 $$\begin{aligned} \Upsilon _{i} =A_{i}^{T}P_{2}+P_{2}^{T}A_{i}-E^{T}W_{1}E<0 \end{aligned}$$

Then, pre- and post-multiplying $\Upsilon _{i}<0$ by $H^{T}$ and *H*, respectively, (16) yields

17 $$\begin{aligned} \tilde{\Upsilon }_{i}=\tilde{A}_{i}^{T}\tilde{P}+\tilde{P}^{T}\tilde{A}_{i}-H^{T}E^{T}W_{1}EH =\begin{bmatrix} \tilde{\Upsilon }_{11}&\quad \tilde{\Upsilon }_{12} \\ *&\quad \tilde{A}_{i22}^{T}\tilde{P}_{22}+\tilde{P}^{T}_{22}\tilde{A}_{i22} \end{bmatrix}<0 \end{aligned}$$

Since $\tilde{\Upsilon }_{11}$ and $\tilde{\Upsilon }_{12}$ are irrelevant to the results of the following discussion, the real expression of these two variables are omitted here. From Eq. (17), it is easy to see that

$$\begin{aligned} \tilde{A}_{i22}^{T}\tilde{P}_{22}+\tilde{P}^{T}_{22}\tilde{A}_{i22}<0 \end{aligned}$$

Since $\mu _{i}(\xi (t))\ge 0$ and $\sum _{i=1}^{r}\mu _{i}(\xi (t))=1$, we have

$$\begin{aligned} \sum _{i=1}^{r}\mu _{i}(\xi (t))(\tilde{A}_{i22}^{T} \tilde{P}_{22}+\tilde{P}^{T}_{22}\tilde{A}_{i22})<0 \end{aligned}$$

This implies that $\sum _{i=1}^{r}\mu _{i}(\xi (t))\tilde{A}_{i22}$ is nonsingular. Therefore, the unforced fuzzy singular system (5) is regular and impulse free.

Next, we will show the stability of the system (5). Similar to (14)–(15), we define

18 $$\begin{aligned} GA_{\tau i}H&= \begin{bmatrix} \tilde{A}_{\tau i,11}&\quad \tilde{A}_{\tau i,12} \\ \tilde{A}_{\tau i,21}&\quad \tilde{A}_{\tau i,22} \end{bmatrix},\quad G^{-T}W_{1}G^{-1}=\begin{bmatrix} \tilde{W}_{1,11}&\quad \tilde{W}_{1,12} \\ \tilde{W}_{1,21}&\quad \tilde{W}_{1,22} \end{bmatrix} \\ H^{T}Q_{1}H&=\begin{bmatrix} \tilde{Q}_{1,11}&\quad \tilde{Q}_{1,12} \\ \tilde{Q}_{1,21}&\quad \tilde{Q}_{1,22} \end{bmatrix},\quad G^{-T}Y_{ij}G^{-1}=\begin{bmatrix} \tilde{Y}_{ij,11}&\quad \tilde{Y}_{ij,12} \\ \tilde{Y}_{ij,21}&\quad \tilde{Y}_{ij,22} \end{bmatrix} \\ H^{T}S_{1}H&= \begin{bmatrix} \tilde{S}_{1,11}&\quad \tilde{S}_{1,12} \\ \tilde{S}_{1,21}&\quad \tilde{S}_{1,22} \end{bmatrix},\quad G^{-T}Z_{ij}G^{-1}=\begin{bmatrix} \tilde{Z}_{ij,11}&\quad \tilde{Z}_{ij,12} \\ \tilde{Z}_{ij,21}&\quad \tilde{Z}_{ij,22} \end{bmatrix} \end{aligned}$$

If condition (6) holds, we have

19 $$\begin{aligned} \sum _{i=1}^{r}\mu _{i}\begin{bmatrix} P_{2}^{T}A_{i}+A_{i}^{T}P_{2}+Q_{1}+S_{1}-E^{T}W_{1}E&\quad P_{2}^{T}A_{\tau i} \\ *&\quad \Theta _{(N3,3)} \end{bmatrix}<0 \end{aligned}$$

Pre-multiplying and post-multiplying the preceding inequality by $$\begin{bmatrix} H^{T} \ H \end{bmatrix}$$ and its transpose, respectively, since $Q_{1}>0$ and with definitions (18), we can obtain

$$\begin{aligned} \begin{bmatrix} \star&\quad \star&\quad \star&\quad \star \\ \star&\quad \sum _{i=1}^{r}\mu _{i}\Big (\tilde{P}_{22}^{T}\tilde{A}_{i22}+\tilde{A}_{i22}^{T}\tilde{P}_{22} \Big )+\tilde{Q}_{1,22}+\tilde{S}_{1,22}&\quad \star&\quad \tilde{P}_{22}^{T}\sum _{i=1}^{r}\mu _{i}\tilde{A}_{\tau i,22} \\ \star&\quad \star&\quad \star&\quad \star \\ \star&\quad \sum _{i=1}^{r}\mu _{i}\tilde{A}_{\tau i,22}^{T}\tilde{P}_{22}&\quad \star&\quad -(1-d)\tilde{S}_{1,22} \end{bmatrix}<0 \end{aligned}$$

which implies that

20 $$\begin{aligned} \begin{bmatrix} \sum \mu _{i}\Big (\tilde{P}_{22}^{T}\tilde{A}_{i22}+\tilde{A}_{i22}^{T}\tilde{P}_{22} \Big )+\tilde{S}_{1,22}&\quad \tilde{P}_{22}^{T}\sum \mu _{i}\tilde{A}_{\tau i,22} \\ \sum _{i=1}^{r}\mu _{i}\tilde{A}_{\tau i,22}^{T}\tilde{P}_{22}&\quad -(1-d)\tilde{S}_{1,22} \end{bmatrix}<0 \end{aligned}$$

Then, pre-multiplying and post-multiplying (20) by $[-\vartheta ^{T}\ I]$ and its transpose, respectively, (20) yields $\vartheta ^{T}\tilde{S}_{1,22}\vartheta -(1-d)\tilde{S}_{1,22}<0$, which shows that $\rho (\vartheta )<1$ holds for all allowable $\mu _{i}$ with $\vartheta =\big (\sum _{i=1}^{r}\mu _{i}\tilde{A}_{i22}\big )^{-1} \big (\sum _{i=1}^{r}\mu _{i}\tilde{A}_{\tau i22}\big )$.

Then, we define the following Lyapunov–Krasovskii functional for the unforced fuzzy singular system (5),

21 $$\begin{aligned} V(x_{t},t)=V_{1}(t)+V_{2}(t)+V_{3}(t) \end{aligned}$$

where

$$\begin{aligned} V_{1}(t)&= x^{T}(t)E^{T}P_{1}Ex(t) \\ V_{2}(t)&= \sum _{n=1}^{N}\int _{t-nh}^{t-(n-1)h}x^{T}(s)Q_{n}x(s)ds+\int _{t-\tau (t)}^{t}x^{T}(s)S_{1}x(s)ds \\&\quad+\int _{t-\tau _{\rho }}^{t-\tau _{1}} x^{T}(s)S_{2}x(s)ds +\int _{t-\tau _{2}}^{t-\tau _{\rho }} x^{T}(s)S_{3}x(s)ds \\ V_{3}(t)&=\sum _{n=1}^{N}\int _{-nh}^{-(n-1)h}\int _{t+\theta }^{t} {\dot{x}}^{T}(s)hE^{T}W_{n}E{\dot{x}}(s)dsd\theta \\&\quad+\int _{-\tau _{\rho }}^{-\tau _{1}}\int _{t+\theta }^{t}{\dot{x}}^{T}(s) E^{T}R_{1}E{\dot{x}}(s)dsd\theta +\int _{-\tau _{2}}^{-\tau _{\rho }}\int _{t+\theta }^{t}{\dot{x}}^{T}(s) E^{T}R_{2}E{\dot{x}}(s)dsd\theta \end{aligned}$$

where the unknown matrices $P_{1}>0$, $S_{1}>0$, $S_{2}>0$, $S_{3}>0$, $R_{1}>0$, $R_{2}>0$, $Q_{n}>0$ and $W_{n}>0(n=1,2,\ldots ,N)$ are to be determined. Here, in order to reduce the conservativeness and give a set of strict LMIs stability conditions for system (5), we rewrite (5) as follows:

22 $$\begin{aligned} \left\{ \begin{aligned} &{{\bar{E}}}{\dot{{\bar{x}}}}(t)= \bar{A}(t){{\bar{x}}}(t)+\bar{A}_{\tau }(t){{\bar{x}}}(t-\tau (t)) \\ &{{\bar{x}}}(t)= \bar{\phi }(t), t\in [-\tau _{2},0] \end{aligned}\right. \end{aligned}$$

where

$${\bar{E}}=\begin{bmatrix}E&\quad 0 \\ 0&\quad 0 \end{bmatrix}, \bar{A}(t)=\begin{bmatrix}0&\quad I \\ A(t)&\quad -I \end{bmatrix}, \bar{A}_{\tau }(t)=\begin{bmatrix}0&\quad 0 \\ A_{\tau }(t)&\quad 0 \end{bmatrix},{\bar{x}}(t)=\begin{bmatrix} x(t) \\ E{\dot{x}}(t) \end{bmatrix}$$.

Then, we have

$$\begin{aligned} V_{1}(t)=x^{T}(t)(E^{T}P_{1}E+E^{T}RS^{T})x(t)= {{\bar{x}}}^{T}(t){\bar{E}}^{T}\bar{P}{{\bar{x}}}(t) \end{aligned}$$

where $$\bar{P}=\begin{bmatrix}P_{1}E+RS^{T}&0 \\ P_{2}&P_{3} \end{bmatrix}$$ with $${\bar{E}}^{T}\bar{P}=\bar{P}^{T}{\bar{E}}$$.

Therefore, the time derivatives of $V_{1}(t)$ along the trajectories of the systems (22) satisfies

$$\begin{aligned} \dot{V}_{1}(t)&= {{\bar{x}}}^{T}(t)[\bar{P}^{T}\bar{A}(t)+\bar{A}^{T}(t)\bar{P}] {{\bar{x}}}(t)+ 2{\bar{x}}^{T}(t)\bar{P}^{T}\bar{A}_{\tau }(t){\bar{x}}(t-\tau (t))\\&= \begin{bmatrix} x^{T}(t)&\quad x^{T}(t-\tau (t))&\quad (E{\dot{x}}(t))^{T} \end{bmatrix} \Phi (t) \begin{bmatrix} x(t) \\ x(t-\tau (t)) \\ E{\dot{x}}(t) \end{bmatrix} \\ \end{aligned}$$

where

$$\begin{aligned} \Phi (t)=\begin{bmatrix} A(t)P_{2}+P_{2}A(t)&\quad P_{2}A_{\tau }(t)&\quad E^{T}P_{1}+SR^{T}-P^{T}_{2}+A^{T}(t)P_{3} \\ \star&\quad 0&\quad A^{T}_{\tau }(t)P_{3} \\ \star&\quad \star&\quad -P^{T}_{3}-P_{3} \end{bmatrix} \end{aligned}$$

Similarly, $V_{2}(t)$ and $V_{3}(t)$ along the trajectories of the systems (5) satisfy

23 $$\begin{aligned} \dot{V}_{2}(t)&=\sum _{n=1}^{N}x^{T}(t-(n-1)h)Q_{n}x(t-(n-1)h) \\&\quad -\sum _{n=1}^{N}x^{T}(t-nh)Q_{n}x(t-nh)+x^{T}(t)S_{1}x(t) \\&\quad-(1-\dot{\tau }(t))x^{T}(t-\tau (t))S_{1}x(t-\tau (t))+x^{T}(t-\tau _{1})S_{2}x(t-\tau _{1}) \\&\quad - x^{T}(t-\tau _{\rho })S_{2}x(t-\tau _{\rho }) +x^{T}(t-\tau _{\rho })S_{3}x(t-\tau _{\rho })-x^{T}(t-\tau _{2})S_{3}x(t-\tau _{2}) \\ \end{aligned}$$

24 $$\begin{aligned}\dot{V}_{3}(t)&= {\dot{x}}^{T}(t)E^{T} \left(\sum _{n=1}^{N}h^{2}W_{n}+\rho \delta R_{1}+(1-\rho )\delta R_{2}\right)E {\dot{x}}(t) \\&\quad- \sum _{n=1}^{N}\int _{t-nh}^{t-(n-1)h}{\dot{x}}^{T}(s)hE^{T}W_{n}E{\dot{x}}(s)ds \\&\quad-\int _{t-\tau _{\rho }}^{t-\tau _{1}}{\dot{x}}^{T}(s)E^{T}(R_{1}-Y_{33})E{\dot{x}}(s)ds-\int _{t-\tau _{\rho }}^{t-\tau _{1}}{\dot{x}}^{T}(s)E^{T}Y_{33}E{\dot{x}}(s)ds \\&\quad-\int _{t-\tau _{2}}^{t-\tau _{\rho }}{\dot{x}}^{T}(s)E^{T}(R_{2}-Z_{33})E{\dot{x}}(s)ds -\int _{t-\tau _{2}}^{t-\tau _{\rho }}{\dot{x}}^{T}(s)E^{T}Z_{33}E{\dot{x}}(s)ds \end{aligned}$$

For convenience of notations, in the sequel, we denote $\hat{Y}_{ij}=E^{T}Y_{ij}E$, $\hat{Z}_{ij}=E^{T}Z_{ij}E$. Then, for the Case I, when $\tau _{1} \le \tau (t) \le \tau _{\rho }$, the following equations are true:

25 $$\begin{aligned}&- \int _{t-\tau _{\rho }}^{t-\tau _{1}}{\dot{x}}^{T}(s)\hat{Y}_{33}{\dot{x}}(s)ds -\int _{t-\tau _{2}}^{t-\tau _{\rho }}{\dot{x}}^{T}(s)\hat{Z}_{33}{\dot{x}}(s)ds \nonumber \\&\quad = -\int _{t-\tau _{\rho }}^{t-\tau (t)}{\dot{x}}^{T}(s)\hat{Y}_{33}{\dot{x}}(s)ds-\int _{t-\tau (t)}^{t-\tau _{1}}{\dot{x}}^{T}(s)\hat{Y}_{33}{\dot{x}}(s)ds\nonumber \\&\qquad -\int _{t-\tau _{2}}^{t-\tau _{\rho }}{\dot{x}}^{T}(s)\hat{Z}_{33}{\dot{x}}(s)ds \end{aligned}$$

By utilizing Lemma 3 and the Leibniz–Newton formula, we have

26 $$\begin{aligned} -\int _{t-\tau _{\rho }}^{t-\tau (t)}{\dot{x}}^{T}(s)\hat{Y}_{33}{\dot{x}}(s)ds&\le x^{T}(t-\tau (t))\left[\rho \delta \hat{Y}_{11}+\hat{Y}_{13}+\hat{Y}_{13}^{T}\right] x(t-\tau (t)) \\&\quad + 2x^{T}(t-\tau (t))\left[\rho \delta \hat{Y}_{12}-\hat{Y}_{13}+\hat{Y}_{23}^{T}\right]x(t-\tau _{\rho }) \\&\quad +x^{T}(t-\tau _{\rho })\left[\rho \delta \hat{Y}_{22}-\hat{Y}_{23}-\hat{Y}_{23}^{T}\right]x(t-\tau _{\rho }) \end{aligned}$$

Similarly, we obtain

27 $$\begin{aligned} - \int _{t-\tau (t)}^{t-\tau _{1}}{\dot{x}}^{T}(s)\hat{Y}_{33}{\dot{x}}(s)ds&\le x^{T}(t-\tau _{1})\left[\rho \delta \hat{Y}_{11}+\hat{Y}_{13}+\hat{Y}_{13}^{T}\right]x(t-\tau _{1}) \\&\quad +2x^{T}(t-\tau _{1})\left[\rho \delta \hat{Y}_{12}-\hat{Y}_{13}+\hat{Y}_{23}^{T}\right] x(t-\tau (t)) \\&\quad +x^{T}(t-\tau (t))\left[\rho \delta \hat{Y}_{22}-\hat{Y}_{23}-\hat{Y}_{23}^{T}\right] x(t-\tau (t)) \end{aligned}$$

28 $$\begin{aligned} - \int _{t-\tau _{2}}^{t-\tau _{\rho }}{\dot{x}}^{T}(s)\hat{Z}_{33}{\dot{x}}(s)ds & \le x^{T}(t-\tau _{\rho }) \left[(1-\rho )\delta \hat{Z}_{11}+\hat{Z}_{13}+\hat{Z}_{13}^{T}\right] x(t-\tau _{\rho }) \\&+2x^{T}(t-\tau _{\rho })\left[(1-\rho )\delta \hat{Z}_{12}-\hat{Z}_{13}+\hat{Z}_{23}^{T}\right] x(t-\tau _{2}) \\&+x^{T}(t-\tau _{2}) \left[(1-\rho )\delta \hat{Z}_{22}-\hat{Z}_{23}-\hat{Z}_{23}^{T}\right] x(t-\tau _{2}) \end{aligned}$$

Substituting (25)–(28) into (24), by Lemma 4, a straightforward computation gives

29 $$\begin{aligned} \dot{V}(t)& \le \zeta ^{T}(t)\Theta (t)\zeta (t) -\int _{t-\tau _{\rho }}^{t-\tau _{1}}{\dot{x}}^{T}(s)E^{T}(R_{1}-Y_{33})E{\dot{x}}(s)ds \\&- \int _{t-\tau _{2}}^{t-\tau _{\rho }}{\dot{x}}^{T}(s)E^{T}(R_{2}-Z_{33})E{\dot{x}}(s)ds \end{aligned}$$

where $\zeta ^{T}(t)=[x^{T}(t) \ x^{T}(t-h) \ \cdots \ x^{T}(t-\tau _{1}) \ x^{T}(t-\tau _{\rho }) \ x^{T}(t-\tau (t)) \ x^{T}(t-\tau _{2}) \ (E{\dot{x}}(t))^{T}]$. When $R_{1}-Y_{33}\ge 0$, $R_{2}-Z_{33} \ge 0$, and $\tau _{1}\le \tau (t) \le \tau _{\rho }$, the last two terms in (29) are all less than 0. Therefore, if the conditions (6)–(7) hold, there exist $\alpha >0$ such that $\dot{V}(x_{t})<\alpha \Vert x_{t}\Vert$. By Lemma 6, we conclude that the unforced fuzzy singular system (5) is stable.

For the Case II, when $\tau _{\rho } \le \tau (t) \le \tau _{2}$, the following equations are true:

$$\begin{aligned}&-\int _{t-\tau _{\rho }}^{t-\tau _{1}}{\dot{x}}^{T}(s)\hat{Y}_{33}{\dot{x}}(s)ds -\int _{t-\tau _{2}}^{t-\tau _{\rho }}{\dot{x}}^{T}(s)\hat{Z}_{33}{\dot{x}}(s)ds\\&\quad =-\int _{t-\tau _{\rho }}^{t-\tau _{1}}{\dot{x}}^{T}(s)\hat{Y}_{33}{\dot{x}}(s)ds -\int _{t-\tau _{2}}^{t-\tau (t)}{\dot{x}}^{T}(s)\hat{Z}_{33}{\dot{x}}(s)ds-\int _{t-\tau (t)}^{t-\tau _{\rho }}{\dot{x}}^{T}(s)\hat{Z}_{33}{\dot{x}}(s)ds \end{aligned}$$

Then, the proof can be completed in a similar formulation to Case I and is omitted here for simplification. Therefore, if LMIs (6)–(7) hold, the fuzzy singular system (5) is admissible for the Cases I and II, respectively. This completes the proof. $\square$

For uncertain T–S fuzzy system of (5), the following result can be easily derived by applying Lemma 5 and Schur complement.

#### **Corollary 8**

*For the given scalars*$\tau _{1}$, $\tau _{2}$, *dand*$\rho$, *the uncertain fuzzy system of* (5) *is robustly admissible for any time-varying delay*$\tau (t)$*satisfying* (3), *if there exist matrices*$P_{1}>0$, $Q_{n}>0$, $W_{n}>0\;(n=1,2,\ldots ,N)$, $\Lambda ^{T}(Y_{ij})_{3\times 3}\Lambda =\hat{Y}\ge 0$, $\Lambda ^{T}(Z_{ij})_{3\times 3}\Lambda =\hat{Z}\ge 0$, $\Lambda ={\mathrm{diag}}\{E,E,E\}$, $S_{1}>0$, $S_{2}>0$, $S_{3}>0$, $R_{1}>0$, $R_{2}>0$, *and positive scalars*$\varepsilon _{1i}$, *some appropriate dimension matricesS*, $P_{2}$, $P_{3}$*and the constant matrixRsatisfying*$E^{T}R=0$*such that the following set of LMIs hold:*

30 $$\begin{aligned}&\bar{\Theta }^{i}=\begin{bmatrix} \Theta ^{i}&\quad \Gamma _{1i}^{T}&\quad \Omega _{1i}^{T} \\ *&\quad -\varepsilon _{i}I&\quad 0 \\ *&\quad *&\quad -\varepsilon _{i}I \end{bmatrix} < 0 \end{aligned}$$

31 $$\begin{aligned}&R_{1}-Y_{33}\ge 0, \quad R_{2}-Z_{33}\ge 0 \end{aligned}$$

*with*

$$\begin{aligned} \Omega _{1i}&=[N_{1i} \quad 0 \quad \cdots \quad 0 \quad 0 \quad 0 \quad N_{2i} \quad 0 \quad 0 ] \\ \Gamma _{1i}&=[M_{i}^{T}P_{2} \quad 0 \quad \cdots \quad 0 \quad 0 \quad 0 \quad 0 \quad 0 \quad M_{i}^{T}P_{3} ] \\ \end{aligned}$$

*and*$\Theta ^{i}$*are defined in Theorem* 7.

#### *Remark 9*

Different from the results in Yang et al. (2015), Zeng et al. (2014), Souza et al. (2014), Peng et al. (2011), Peng and Fei (2013), Liu et al. (2010) and An and Wen (2011), by dividing the constant part of time-varying delay $[0, \tau _{1}]$ into *N* segments, that is, $[0,\frac{1}{N}\tau _{1}]$, $[\frac{1}{N}\tau _{1}, \frac{2}{N}\tau _{1}]$, $\cdots$, $[\frac{N-1}{N}\tau _{1},\tau _{1}]$, we define different energy functional $Q_{n}(n=1,2,\ldots ,N)$ in each different delay subinterval segment. Because the piecewise Lyapunov function candidates are much richer than the globally quadratic functions, so the obtained stability criteria based on this method can further reduce the conservativeness of analysis and synthesis.

#### *Remark 10*

Since the interval $[\tau _{1}, \tau _{2}]$ is divided into two unequal variable subintervals $[\tau _{1},\tau _{\rho }]$ and $[\tau _{\rho },\tau _{2}]$ in which $\rho$ is a tunable parameter, it is clear that the LKF defined in Theorem 7 is more general and simple than Zhang et al. (2009) and Mourad et al. (2013) by seeking a appropriate $\rho$ satisfying $0<\rho <1$. For different $\rho$, the LKF matrices may be different and the LMIs also may be different in stability conditions, and thus compared with the methods using the same LKF matrices (Wang et al. 2014) or the uniformly dividing delay subintervals (Yang et al. 2015), the variable and different LKF matrices may lead to less conservativeness.

#### *Remark 11*

The decomposition method in Theorem 7 may increase the maximum allowable upper bounds on $\tau _{2}$ for the fixed lower bound $\tau _{1}$, if one can set a suitable dividing point with relation to $\rho$. For seeking an appropriate $\rho$, a algorithm is given as follows:

Step 1: For the given *d*, choose upper bound on $\delta$ satisfying (6)–(7), select this upper bound as initial value $\delta _{0}$ of $\delta$.
Step 2: Set step lengths, $\delta _{step}$ and $\rho _{step}$ for $\delta$ and $\rho$, respectively. Set *k* as a counter and choose $k=1$. Meanwhile, let $\delta =\delta _{0}+\delta _{step}$ and the initial value $\rho _{0}$ of $\rho$ equals $\rho _{step}$.
Step 3: Let $\rho =k\rho _{step}$, if (6)–(7) are feasible, go to Step 4; otherwise, go to Step 5.
Step 4: Let $\delta _{0}=\delta$, $\rho _{0}=\rho$, $k=1$ and $\delta =\delta _{0}+\delta _{step}$, go to Step 3.
Step 5: Let $k=k+1$, if $k\rho _{step}<1$, then go to Step 3. otherwise, stop.

#### *Remark 12*

In order to further reduce the enlargement of the derivative of LKF, inspired by Liu (2013), a new integral inequality is employed to estimate the integral term, which will be helpful to increase the maximum admissible upper bound of time delay. Moreover, when the information of the time-derivative of delay is unknown or the time delay is not differentiable, just let $S_{1}=0$ and proceed in a similar way as the previous proof, some new stability criteria can be obtained from Theorem 7. Due to limited space, no more tautology here.

### Fuzzy controller design

In this section, based on Theorem 7, we will proposed a design method of fuzzy controller. Consider the controller gain variations might be caused by the inaccuracies of controller implementation, we employ the following controller form with PDC scheme:

**Controller rule***i*: **IF**$\xi _{1}(t)$ is $M_{i1}$ and $\ldots$ and $\xi _{p}(t)$ is $M_{ip}$**THEN**

32 $$\begin{aligned} u(t)=(K_{i}+\Delta K_{i}(t))x(t),\quad i=1,2,\ldots ,r. \end{aligned}$$

where $K_{i}$ are the local gain matrices to be determined, and $\Delta K_{i}(t)$ is the controller gain perturbations and satisfies

33 $$\begin{aligned} \Delta K_{i}(t)=M_{ai}F_{a}(t)N_{ai} \end{aligned}$$

where $M_{ai}$ and $N_{ai}$ are known matrices, and $F_{a}(t)$ is an unknown time-varying matrix satisfying $F_{a}^{T}(t)F_{a}(t)\le I$.

Then, the resulting closed-loop system from (1) and (32) can be written as

34 $$\begin{aligned} E{\dot{x}}(t)=&\sum _{i=1}^{r}\sum _{j=1}^{r}\mu _{i}(\xi (t))\mu _{j}(\xi (t))\{ ((A_{i}+\Delta A_{i}(t))\\&+B_{i}(K_{j}+\Delta K_{j}(t)))x(t) +(A_{\tau i}+\Delta A_{\tau i}(t))x(t-\tau (t)) \} \end{aligned}$$

The aim of this section is to design a state feedback controller in the form of (32) with the gain perturbations satisfying (33), such that the closed-loop system (34) is regular, impulse-free, and asymptotically stable.

#### **Theorem 13**

*For the given scalars*$$\tau _{1}$$, $$\tau _{2}$$, *dand tuning parameter*$$\rho$$, *the closed-loop fuzzy singular system* (34) *under fuzzy control* (32) *is robustly admissible for any time-varying delay*$$\tau (t)$$*satisfying* (3), *if there exist matrices*$$X>0$$, $$V_{j}$$, $$\bar{P}_{1}>0$$, $$\bar{Q}_{n}>0$$, $$\bar{W}_{n}>0$$, $$\tilde{W}_{n}>0$$$$(n=1,2,\ldots ,N)$$, $$\bar{S}_{1}>0$$, $$\bar{S}_{2}>0$$, $$\bar{S}_{3}>0$$, $$\bar{R}_{1}>0$$, $$\bar{R}_{2}>0$$, $$\bar{Y}=(\bar{Y}_{ij})_{3\times 3}\ge 0$$, $$\bar{Z}=(\bar{Z}_{ij})_{3\times 3}\ge 0$$, *some positive scalars*$$\varepsilon _{1i}$$, $$\varepsilon _{2ij}$$*and any matrices*$$\bar{S}$$, $$\bar{P}_{2}$$, $$\bar{R}$$*with appropriate dimension such that the following set of LMIs hold for*$$i,j=1,2,\ldots ,r$$:

35 $$\begin{aligned} \begin{bmatrix} \bar{\Xi }_{1}^{ij}&\quad \bar{\Xi }_{2}^{ij}\\ *&\quad \bar{\Xi }_{3}^{ij} \end{bmatrix} < 0 \end{aligned}$$

*and*

36 $$\begin{aligned} \bar{R}_{1}-\bar{Y}_{33}\ge 0, \quad \bar{R}_{2}-\bar{Z}_{33}\ge 0 \end{aligned}$$

*where*

37 $$\begin{aligned} \bar{\Xi }_{1}^{ij}&=\begin{bmatrix} \bar{\Xi }_{11}^{ij}&\quad \bar{\Xi }_{12}^{ij} \ \\ *&\quad \bar{\Xi }_{22}^{i} \ \end{bmatrix} \end{aligned}$$

38 $$\begin{aligned} \bar{\Xi }_{11}^{ij}&=\begin{bmatrix} \bar{\Xi }_{1,1}^{ij}&\quad \bar{W}_{1}&\quad \cdots&\quad 0 \\ *&\quad \bar{\Xi }_{2,2}&\quad \cdots&\quad 0 \\ \vdots&\quad \vdots&\quad \ddots&\quad \vdots \\ *&\quad *&\quad \cdots&\quad \bar{\Xi }_{n,n} \\ \end{bmatrix} \end{aligned}$$

39 $$\begin{aligned} \bar{\Xi }_{12}^{ij}&=\begin{bmatrix} 0&\quad 0&\quad \bar{\Xi }_{(1,N3)}^{i}&\quad 0&\quad \bar{\Xi }_{(1,N5)}^{ij} \\ 0&\quad 0&\quad 0&\quad 0&\quad 0 \\ \vdots&\quad \vdots&\quad \vdots&\quad \vdots&\quad \vdots \\ \bar{W}_{N}&\quad 0&\quad 0&\quad 0&\quad 0 \\ \end{bmatrix} \end{aligned}$$

40 $$\begin{aligned} \bar{\Xi }_{22}^{i}&=\begin{bmatrix} \bar{\Xi }_{(N1,1)}&\quad \bar{\Xi }_{(N1,2)}&\quad \bar{\Xi }_{(N1,3)}&\quad 0&\quad 0 \\ *&\quad \bar{\Xi }_{(N2,2)}&\quad \bar{\Xi }_{(N2,3)}&\quad \bar{\Xi }_{(N2,4)}&\quad 0 \\ *&\quad *&\quad \bar{\Xi }_{(N3,3)}&\quad \bar{\Xi }_{(N3,4)}&\quad \bar{\Xi }_{(N3,5)}^{i} \\ *&\quad *&\quad *&\quad \bar{\Xi }_{(N4,4)}&\quad 0 \\ *&\quad *&\quad *&\quad *&\quad \bar{\Xi }_{(N5,5)} \end{bmatrix} \end{aligned}$$

41 $$\begin{aligned} \bar{\Xi }_{2}^{ij}&=[\varepsilon _{1i}\bar{\Gamma }_{1i} \quad \bar{\Omega }_{1i}^{T}\quad \varepsilon _{2ij}\bar{\Gamma }_{2ij}\quad \bar{\Omega }_{2j}^{T}] \nonumber \\ \bar{\Xi }_{3}^{ij}&={\mathrm{diag}}\{-\varepsilon _{1i}I \ \ -\varepsilon _{1i}I \ \ -\varepsilon _{2ij}I \ \ -\varepsilon _{2ij}I \} \end{aligned}$$

*with*

42 $$\begin{aligned}&\bar{\Xi }_{1,1}^{ij}=A_{i}X+B_{i}V_{j}+(A_{i}X+B_{i}V_{j})^{T}+\bar{Q}_{1}+\bar{S}_{1}-\bar{W}_{1} \\&\bar{\Xi }_{n,n}=-\bar{Q}_{n-1}-\bar{W}_{n-1}+\bar{Q}_{n}-\bar{W}_{n}, \ n=2,3,\ldots ,N \\&\Xi _{(1,N3)}^{i}=A_{\tau i}X, \ \Xi _{(1,N5)}^{ij}=\bar{P}_{1}+\bar{S}\bar{R}^{T}-X+\lambda (A_{i}X+B_{i}V_{j})^{T} \\&\bar{\Xi }_{(N1,1)}=-\bar{Q}_{N}-\bar{W}_{N}+\bar{S}_{2}+\rho \delta \bar{Y}_{11}+\bar{Y}_{13}+\bar{Y}_{13}^{T} \\&\bar{\Xi }_{(N2,2)}=\bar{S}_{3}-\bar{S}_{2}+\rho \delta \bar{Y}_{22}-\bar{Y}_{23}-\bar{Y}_{23}^{T} +(1-\rho )\delta \bar{Z}_{11}+\bar{Z}_{13}+\bar{Z}_{13}^{T} \\&\bar{\Xi }_{(N3,5)}^{i}=\lambda X^{T}A_{\tau i}^{T}, \ \bar{\Xi }_{(N4,4)}=-\bar{S}_{2}+(1-\rho ) \delta \bar{Z}_{22}-\bar{Z}_{23}-\bar{Z}_{23}^{T} \\&\bar{\Xi }_{(N5,5)}=h^2\tilde{W}_{n}+\rho \delta R_{1}+(1-\rho \delta )R_{2}-\lambda \left(X+X^{T}\right) \end{aligned}$$

43 $$\begin{aligned}&\bar{\Gamma }_{1i}=[M_{i}^{T} \quad 0 \quad \cdots \quad 0 \quad 0 \quad 0 \quad 0 \quad 0 \quad \lambda M_{i}^{T} ]^{T} \\&\bar{\Gamma }_{2ij}=[M_{aj}^{T}B_{i}^{T} \quad 0 \quad \cdots \quad 0 \quad 0 \quad 0 \quad 0 \quad 0 \quad \lambda M_{aj}^{T}B_{i}^{T} ]^{T} \\&\bar{\Omega }_{1i}=[N_{1i}X \quad 0 \quad \cdots \quad 0 \quad 0 \quad 0 \quad N_{2i}X \quad 0 \quad 0 ] \\&\bar{\Omega }_{2j}=[N_{aj}X \quad 0 \quad \cdots \quad 0 \quad 0 \quad 0 \quad 0 \quad 0 \quad 0 ] \end{aligned}$$

***Case I:****when*$$\tau _{1} \le \tau (t) \le \tau _{\rho }$$

44 $$\begin{aligned} \bar{\Xi }_{(N1,3)}&=\bar{\Xi }^{T}_{(N2,3)}=\rho \delta \bar{Y}_{12}-\bar{Y}_{13}+\bar{Y}_{23}^{T}, \bar{\Xi }_{(N3,4)}=0 \nonumber \\ \bar{\Xi }_{(N2,4)}&=(1-\rho )\delta \bar{Z}_{12}-\bar{Z}_{13}+\bar{Z}_{23}^{T}, \bar{\Xi }_{(N1,2)}=0 \nonumber \\ \bar{\Xi }_{(N3,3)}&=-(d-1)\bar{S}_{1}+\rho \delta (\bar{Y}_{11}+ \bar{Y}_{22})+\bar{Y}_{13}+\bar{Y}_{13}^{T}-\bar{Y}_{23}-\bar{Y}_{23}^{T} \end{aligned}$$

***Case II:****when*$\tau _{\rho } \le \tau (t) \le \tau _{2}$

45 $$\begin{aligned} \bar{\Xi }_{(N1,2)}&=\rho \delta \bar{Y}_{12}-\bar{Y}_{13}+\bar{Y}_{23}^{T}, \bar{\Xi }_{(N2,4)}= \bar{\Xi }_{(N1,3)}=0\nonumber \\ \bar{\Xi }_{(N2,3)}&=\bar{\Xi }_{(N3,4)}=(1-\rho )\delta \bar{Z}_{12}-\bar{Z}_{13}+\bar{Z}_{23}^{T}\nonumber \\ \bar{\Xi }_{(N3,3)}&=-(1-d)\bar{S}_{1}+(1-\rho )\delta \bar{Z}_{11} +\bar{Z}_{13} +\bar{Z}_{13}^{T}+(1-\rho )\delta \bar{Z}_{22}-\bar{Z}_{23}-\bar{Z}_{23}^{T} \end{aligned}$$

*Moreover, if the aforementioned condition is feasible, the gain matrices of controller in the form of* (32) *can be designed by*$K_{j}=V_{j}X^{-1}$.

#### *Proof*

For the uncertain closed-loop T–S fuzzy singular system (34), replacing $A_{i}$ and $A_{\tau i}$ with $((A_{i}+\Delta A_{i}(t))+B_{i}(K_{j}+\Delta K_{j}(t)))$, $A_{\tau i }+\Delta A_{\tau i}(t)$ in system (5), respectively. Then, according to (2) and (33), the condition (6) can be rewritten as

46 $$\begin{aligned} \Xi ^{ij}_{1}+ \Gamma _{1i}F(t)&\Omega _{1i}+\Omega _{1i}^{T}F^{T}(t)\Gamma _{1i}^{T} +\Gamma _{2ij}F_{a}(t)\Omega _{2j}+\Omega _{2j}^{T}F^{T}_{a}(t)\Gamma _{2ij}^{T}<0 \end{aligned}$$

where $$\Xi ^{ij}_{1}=\begin{bmatrix} \ \Xi _{11}^{ij}&\quad \Xi _{12}^{ij} \ \\ \ *&\quad \Xi _{22}^{i} \ \end{bmatrix}$$ and

47 $$\begin{aligned} \Xi _{11}^{ij}&=\begin{bmatrix} \Xi _{1,1}^{ij}&\quad E^{T}W_{1}E&\quad \cdots&\quad 0 \\ *&\quad \Theta _{2,2}&\quad \cdots&\quad 0 \\ \vdots&\quad \vdots&\quad \ddots&\quad \vdots \\ *&\quad *&\quad \cdots&\quad \Theta _{n,n} \end{bmatrix} \end{aligned}$$

48 $$\begin{aligned} \Xi _{12}^{ij}&=\begin{bmatrix} 0&\quad 0&\quad P_{2}^{T}A_{\tau i}&\quad 0&\quad \Xi _{(1,N5)}^{ij} \\ 0&\quad 0&\quad 0&\quad 0&\quad 0 \\ \vdots&\quad \vdots&\quad \vdots&\quad \vdots&\quad \vdots \\ E^{T}W_{N}E&\quad 0&\quad 0&\quad 0&\quad 0 \\ \end{bmatrix} \end{aligned}$$

49 $$\begin{aligned} \Xi _{22}^{i}&=\begin{bmatrix} \Theta _{(N1,1)}&\quad \Theta _{(N1,2)}&\quad \Theta _{(N1,3)}&\quad 0&\quad 0 \\ *&\quad \Theta _{(N2,2)}&\quad \Theta _{(N2,3)}&\quad \Theta _{(N2,4)}&\quad 0 \\ *&\quad *&\quad \Theta _{(N3,3)}&\quad \Theta _{(N3,4)}&\quad A_{\tau i}^{T}P_{3} \\ *&\quad *&\quad *&\quad \Theta _{(N4,4)}&\quad 0 \\ *&\quad *&\quad *&\quad *&\quad \Theta _{(N5,5)} \end{bmatrix} \end{aligned}$$

with

$$\begin{aligned} \Xi _{1,1}^{ij}&=P_{2}^{T}(A_{i}+B_{i}K_{j})+(A_{i}+B_{i}K_{j})^{T}P_{2} +Q_{1}+S_{1}-E^{T}W_{1}E \\ \Xi _{(1,N5)}^{ij}&=E^{T}P_{1}+SR^{T}-P^{T}_{2}+(A_{i}+B_{i}K_{j})^{T}(t)P_{3} \\ \Omega _{1i}&=\begin{bmatrix}N_{1i}&\quad 0&\quad \cdots&\quad 0&\quad 0&\quad 0&\quad N_{2i}&\quad 0&\quad 0 \end{bmatrix} \\ \Omega _{2j}&=\begin{bmatrix}N_{aj}&\quad 0&\quad \cdots&\quad 0&\quad 0&\quad 0&\quad 0&\quad 0&\quad 0 \end{bmatrix} \\ \Gamma _{1i}&=\begin{bmatrix}M_{i}^{T}P_{2}&\quad 0&\quad \cdots&\quad 0&\quad 0&\quad 0&\quad 0&\quad 0&\quad M_{i}^{T}P_{3} \end{bmatrix}^{T} \\ \Gamma _{2ij}&=\begin{bmatrix}M_{aj}^{T}B_{i}^{T}P_{2}&\quad 0&\quad \cdots&\quad 0&\quad 0&\quad 0&\quad 0&\quad 0&\quad M_{aj}^{T}B_{i}^{T}P_{3} \end{bmatrix}^{T} \end{aligned}$$

and other matrix elements such as $\Theta _{ij}$ are defined in Theorem 7. By Lemma 5, we get from (46) that

50 $$\begin{aligned} \Xi ^{ij}_{1}+\varepsilon _{1i}\Gamma _{1i}\Gamma _{1i}^{T} +\varepsilon ^{-1}_{1i}\Omega _{1i}^{T}\Omega _{1i} +\varepsilon _{2ij}\Gamma _{2ij}\Gamma _{2ij}^{T} +\varepsilon ^{-1}_{2ij}\Omega _{2j}^{T}\Omega _{2j}<0 \end{aligned}$$

where scalars $\varepsilon _{1ij}>0$ and $\varepsilon _{2ij}>0$. Then, by Schur complement, inequality (50) equals to

51 $$\begin{aligned} \begin{bmatrix} \ \Xi _{1}^{ij}&\quad \Xi _{2}^{ij} \ \\ \ *&\quad \Xi _{3}^{ij} \ \end{bmatrix} < 0 \end{aligned}$$

where

$$\begin{aligned}&\Xi _{2}^{ij}=[\varepsilon _{1i}\Gamma _{1i} \quad \Omega _{1i}^{T}\quad \varepsilon _{2ij}\Gamma _{2ij}\quad \Omega _{2j}^{T}] \\&\Xi _{3}^{ij}={\mathrm{diag}}\{-\varepsilon _{1i}I \quad -\varepsilon _{1i}I \quad -\varepsilon _{2ij}I \quad -\varepsilon _{2ij}I \} \end{aligned}$$

In order to obtain the control gain matrix, take $P_{3}=\lambda P_{2}$, where $\lambda$ is the designing parameter and define the following matrices variables:

$$\begin{aligned}&X=P_{2}^{-1},\ V_{j}=K_{j}X, \ X^{T}E^{T}P_{1}X=\bar{P}_{1}, \quad X^{-1}R^{T}X=\bar{R}\\&X^{T}Q_{n}X=\bar{Q}_{n}, X^{T}E^{T}W_{n}EX=\bar{W}_{n}, X^{T}W_{n}X=\tilde{W}_{n} \quad (n=1,2,3,\ldots ,N) \\&X^{T}S_{n}X=\bar{S}_{n},\ X^{T}R_{n}X=\bar{R}_{n}, \quad (n=1,2) \\&X^{T}E^{T}Y_{ij}EX=\bar{Y}_{ij}, \ X^{T}E^{T}Z_{ij}EX=\bar{Z}_{ij}, \quad (i=1,2,3; j=1,2,3) \\ \end{aligned}$$

Then, pre- and post-multiplying both sides of inequality (51) with ${\mathrm{diag}}\{X^{T},\ldots ,X^{T},I,I,I,I\}$ and its transpose, respectively, we can obtain the conditions (35) and (36), which means that the closed-loop fuzzy singular system (34) is regular, impulse-free and stable under fuzzy control (32). This completes the proof. $\square$

#### *Remark 14*

Different from the work in Su et al. (2013) concerned with dynamic output controller design for discrete-time T–S fuzzy delay systems, this study is mainly focused on the state feedback controller design for T–S fuzzy singular systems with time-varying delay while the gain variations may be caused by the inaccuracies of controller implementation. In addition, the input–output technique (Su et al. 2013; Zhao et al. 2013) is employed to reduce the conservativeness in stability analysis, however, the model transformation of the original system will result in approximation error. In this study, only need to select a appropriate $\rho$ in the new constructed LKF, less conservative stability and stabilization conditions can be directly obtained. In Examples 1–3, the comparison results with input–output approach in Su et al. (2013) and other methods to deal with time delays are presented to illustrate the advantages of the proposed approach.

#### *Remark 15*

It should be mentioned that the main character of delay partitioning approach lies in that when the number of subintervals *N* is increased, the conservatism of the result decreases. Meanwhile, the computational complexity increases, see Yang et al. (2015), Wang et al. (2014) and Peng and Fei (2013). Therefore, the choice of the number of subintervals *N* generally depends on the tradeoff between the conservatism reduction and the computational burden. However, according to the examples presented in the next section, we can see that our results ($N=1$) used less partitioning segments is much better than the one in Wang et al. (2014) ($N=2$), Peng and Fei (2013) ($N=3$) and Yang et al. (2015) ($N=3$), It is means that the presented approach has higher computational efficiency, especially when the number of delay partitioning segments is large.

## Numerical examples

In this section, four examples are given to demonstrate the effectiveness of the proposed approaches. The first three examples are presented to show the improvement of our results over the existing ones. The last example is used to demonstrate the applicability of the controller design method.

### *Example 16*

Consider the following time-delayed nonlinear system:

$$\begin{aligned} \left\{ \begin{aligned} &{\dot{x}}_{1}(t)=0.5(1-sin^{2}(\theta (t)))x_{2}(t)-x_{1}(t-\tau (t))-(1+sin^{2}(\theta (t)))x_{1}(t) \\ &{\dot{x}}_{2}(t)=sgn( | \theta (t)|-\frac{\pi }{2})(0.9cos^{2}(\theta (t))-1)x_{1}(t-\tau (t))-x_{2}(t-\tau (t)) \\&\quad\qquad-(0.9+0.1cos^{2}(\theta (t)))x_{2}(t) \end{aligned}\right. \end{aligned}$$

which can be exactly expressed as a nominal T–S delayed system with the following rules:

$$\begin{aligned}&Rule\,1{:}\,if \theta (t)\,is \pm \frac{\pi }{2}, \quad then \ {\dot{x}}(t)=A_{1}x(t)+A_{\tau 1}x(t-\tau (t)) \\&Rule\,2{:}\,if \theta (t)\,is \ 0 , \quad then \ {\dot{x}}(t)=A_{2}x(t)+A_{\tau 2}x(t-\tau (t)) \end{aligned}$$

where the membership functions for above rule 1 and rule 2 are $h_{1}(\theta (t))=sin^{2}(\theta (t))$, $h_{2}(\theta (t))=cos^{2}(\theta (t))$ with $\theta (t)=x_{1}(t)$, and the system matrices are:

$$\begin{aligned} A_{1}=\begin{bmatrix} -2&\quad 0 \\ 0&\quad -0.9 \end{bmatrix}, \quad A_{\tau 1}= \begin{bmatrix} -1&\quad 0 \\ -1&\quad -1 \end{bmatrix},\quad A_{2}=\begin{bmatrix} -1&\quad 0.5 \\ 0&\quad \ -1 \end{bmatrix}, \quad A_{\tau 2}= \begin{bmatrix} -1&\quad 0 \\ 0.1&\quad -1 \end{bmatrix} \end{aligned}$$

For this example, because the time-derivative of delay $\tau (t)$ is unknown and the considered systems is nonsingular, we set $S_{1}=0$, $E=I_{2\times 2}$ in Theorem 7 and choose the delay interval segmentation parameter $\rho =0.7$ in Case I, $\rho = 0.3$ in Case II, respectively. The upper delay bounds $\tau _{2}$ derived by the input–output method (Zhao et al. 2013), convex combination technique (An and Wen 2011; Peng and Fei 2013), free weighting matrices approach (Tian et al. 2009; Souza et al. 2014) and the improved delay partitioning method proposed in this paper are tabulated in Table 1 under different values of $\tau _{1}$. It is seen from Table 1 that the results obtained from Theorem 7 of this paper are significantly better than those obtained from the other methods. When the system matrices of rule 2 are given as Lien et al. (2007) with

$$\begin{aligned} A_{2}=\begin{bmatrix} -1.5&\quad 1 \\ 0&\quad -0.75 \end{bmatrix}, \quad A_{\tau 2}= \begin{bmatrix} -1&\quad 0 \\ 1&\quad -0.85 \end{bmatrix} \end{aligned}$$

the improvement of this paper is shown in Table 2. It can be concluded that the obtained results in our method are less conservative than those of Souza et al. (2014), Peng et al. (2011), Tian et al. (2009), Tian and Chen (2006) and Lien et al. (2007). Moreover, it is shown in Tables 1 and 2 that the conservatism is gradually reduced with the increase of *N* while guaranteeing asymptotically stability of the considered system.

**Table 1:** Example 1-maximum allowable delay bounds of $\tau _{2}$ under different values of $\tau _{1}$ with *d* unknown

Methods${\setminus } \tau _{1}$	0	0.4	0.8	1.0	1.2
Tian et al. (2009) Corollary 1	–	1.2647	1.3032	1.3528	1.4214
An and Wen (2011) Theorem 1	1.2780	1.3030	1.3160	1.3610	1.4250
Souza et al. (2014) Corollary 4	–	1.2836	1.3394	1.4009	1.4815
Peng and Fei (2013) Theorem 1($N=2$)	1.3400	1.3200	1.3200	–	1.4200
Peng and Fei (2013) Theorem 1($N=3$)	1.3800	1.3900	1.4300	–	1.5700
Zhao et al. (2013) Theorem 1	–	1.3802	1.4627	–	1.6066
Xia et al. (2014) Theorem 4	–	1.5274	1.5361	1.5762	1.6340
$C II\,(\rho =0.7, N=1)$	1.4841	1.6743	1.7794	1.7965	1.7805
$C II\,(\rho =0.7, N=2)$	1.4839	1.6761	1.8001	1.8403	1.8699
$C I\,(\rho =0.3, N=1)$	3.2721	2.5582	2.0346	1.8698	1.7495
$C I\,(\rho =0.3, N=2)$	3.2712	2.6034	2.1798	2.0577	1.9769

**Table 2:** Example 1-maximum allowable delay bounds of $\tau _{2}$ under different values of $\tau _{1}$ with *d* unknown

Methods${\setminus } \tau _{1}$	0.2	0.4	0.6	0.8
Tian and Chen (2006)	0.6870	0.8500	0.9460	1.0480
Lien et al. (2007) Corollary 1	0.7945	0.8487	0.9316	1.0325
Peng et al. (2011) Corollary 5	0.9119	0.9793	1.0639	1.1662
Tian et al. (2009) Corollary 1	1.1410	1.1500	1.1720	1.2090
Souza et al. (2014) Corollary 4	1.1639	1.1734	1.1994	1.2532
$C II\,(N=1,\rho =0.95)$	1.3775	1.4419	1.4837	1.5002
$C II\,(N=2,\rho =0.95)$	1.3780	1.4447	1.4940	1.5279
$C I\,(N=1,\rho =0.35)$	2.3328	2.0765	1.8643	1.6990
$C I\,(N=2,\rho =0.35)$	2.3371	2.1024	1.9288	1.8101

### *Example 17*

Consider the following uncertain fuzzy system with two rules:

$$\begin{aligned} {\dot{x}}(t)=\sum _{i=1}^{2}\mu _{i}(\xi (t))\{A_{i}x(t)+A_{\tau i}x(t-\tau (t)) \} \end{aligned}$$

where

$$\begin{aligned} A_{1}&=\begin{bmatrix} -2&\quad 1 \\ 0.5&\quad -0.1 \end{bmatrix},\quad A_{\tau 1}= \begin{bmatrix} -1&\quad 0 \\ -1&\quad -1 \end{bmatrix}, \quad E_{1}=\begin{bmatrix} 1.6&\quad 0 \\ 0&\quad 0.05 \end{bmatrix}\\ A_{2}&=\begin{bmatrix} -2&\quad 0 \\ 0&\quad -1 \end{bmatrix},\quad A_{\tau 2}= \begin{bmatrix} -1.6&\quad 0 \\ 0&\quad -1 \end{bmatrix},\quad E_{\tau 1}= \begin{bmatrix} 0.1&\quad 0 \\ 0&\quad 0.3 \end{bmatrix} \\ E_{2}&=\begin{bmatrix} 1.6&\quad 0 \\ 0&\quad -0.05 \end{bmatrix},\quad E_{\tau 2}= \begin{bmatrix} 0.1&\quad 0 \\ 0&\quad 0.3 \end{bmatrix},\quad D=\begin{bmatrix} 0.03&\quad 0 \\ 0&\quad -0.03 \end{bmatrix} \end{aligned}$$

and the membership functions for rules 1 and 2 are the same as Example 16. For various *d*, by utilizing Corollary 8 and the conditions in Yang et al. (2015), Zeng et al. (2014), Liu et al. (2010) and Lien et al. (2007), the computed upper bounds that guarantee the robust stability of the considered system are summarized in Table 3. It can be concluded that the result proposed in this paper is better than the aforementioned results. In addition, compared with the results in Yang et al. (2015), assume that $i=2$, there are ($13n(n+1)/2)+7n^{2}$ ($N=3$) scalar decision variables and six LMIs in their Theorem 1. However, different from the delay interval $[\tau _{1},\tau _{2}]$ is divided into multiple segments, we divide the delay interval into two unequal subintervals by seeking a appropriate $\rho$. Thus, only $(10n(n+1)/2)+4n^{2}$ ($N=1$) scalar decision variables and four LMIs are required to improve the results. Especially, when *N* is increased, less number of decision variables and LMIs may reduce the mathematical complexity and computational load.

**Table 3:** Example 2-maximum allowable delay bounds of $\tau _{2}$ for different values of *d* with $\tau _{1}=0$

Methods$\setminus d$	0	0.01	0.1	0.5	Unknown
Lien et al. (2007) Theorem 1	1.1680	1.1630	1.1220	0.9340	0.4990
Liu et al. (2010) Corollary 4	1.1920	1.1870	1.1550	1.1000	1.0500
Zeng et al. (2014) Theorem 1 ($m=2$)	1.3900	1.3820	1.3180	1.1320	1.1270
Yang et al. (2015) Theorem 1 ($m=2$)	1.4737	–	1.4182	1.2916	1.2299
Yang et al. (2015) Theorem 1 ($m=3$)	1.6425	–	1.5990	1.4923	1.4182
$C I\,(\rho =0.45, N=1)$	2.7084	2.6940	2.6007	2.2059	1.8245

**Table 4:** Example 3-maximum allowable delay bounds of $\tau _{2}$ for different values of *d* with $\tau _{1}=2$

Methods${\setminus } d$	0.1	0.35	0.6	0.85	0.9	0.95
Zhang et al. (2009) Theorem 1	3.3623	2.9810	2.6010	1.8330	1.0380	–
Mourad et al. (2013) Theorem 3	3.3685	3.1560	3.1510	3.0760	2.6750	2.0780
Wang et al. (2014) Theorem 1 ($N=1$)	3.5023	3.2915	3.2379	3.1321	3.9775	2.5863
Wang et al. (2014) Theorem 1 ($N=2$)	3.6761	3.4755	3.3580	3.2425	3.0737	2.8257
$C II\,(\rho =0.95, N=1)$	3.7445	3.7553	3.7566	3.7573	3.7550	3.7546
$C II\,(\rho =0.95, N=2)$	3.8070	3.8066	3.8064	3.8045	3.8065	3.8072
$C I\,(\rho =0.45, N=1)$	5.2484	4.4255	4.1246	4.0834	4.0663	4.0552
$C I\,(\rho =0.45, N=2)$	5.3596	4.5894	4.3146	4.2472	4.2140	4.1870

### *Example 18*

Consider a continuous fuzzy singular system composed of two rules and the following system matrices:

$$\begin{aligned} E= & {} \begin{bmatrix} 1&\quad 0&\quad 0&\quad 0\\ 0&\quad 1&\quad 0&\quad 0\\ 0&\quad 0&\quad 1&\quad 0\\ 0&\quad 0&\quad 0&\quad 0 \end{bmatrix},\quad A_{1}=\begin{bmatrix} -3&\quad 0&\quad 0&\quad 0.2\\ 0&\quad -4&\quad 0.1&\quad 0\\ 0&\quad 0&\quad -0.1&\quad 0\\ 0.1&\quad 0.1&\quad -0.2&\quad -0.2 \end{bmatrix},\quad A_{\tau 1}=\begin{bmatrix} -0.5&\quad 0&\quad 0&\quad 0\\ 0&\quad -1&\quad 0&\quad 0\\ 0&\quad 0.1&\quad -0.2&\quad 0\\ 0&\quad 0&\quad 0&\quad 0 \end{bmatrix}\\ A_{2}= & {} \begin{bmatrix} -2&\quad 0&\quad 0&\quad -0.2\\ 0&\quad -2.5&\quad -0.1&\quad 0\\ 0&\quad -0.2&\quad -0.3&\quad 0\\ 0.1&\quad 0.1&\quad -0.2&\quad -0.2 \end{bmatrix},\quad A_{\tau 2}=\begin{bmatrix} -0.5&\quad 0&\quad 0&\quad 0\\ 0&\quad -1&\quad 0&\quad 0\\ 0&\quad 0.1&\quad -0.5&\quad 0\\ 0&\quad 0&\quad 0&\quad 0 \end{bmatrix} \end{aligned}$$

In order to compare with the existing results, supposing that $\tau (t)$ satisfies (3) and with $\tau _{1}=2$. Then, setting $\rho =0.45$, $\rho =0.95$ in Cases I and II, respectively. Table 4 presents a comparison results with various *d*, which show that the stability condition in Theorem 7 give less conservative results than those in Wang et al. (2014), Mourad et al. (2013) and Zhang et al. (2009). It is worth mention that the stability conditions in the aforementioned works are not in strict LMIs form due to equality constraints. However, by introducing the variable *R*, much better results are obtained by solving strict LMIs via the existing numerical convex optimization method.

### *Example 19*

Consider the following nonlinear time-delay systems borrowed from Lin et al. (2006):

$$\begin{aligned} (1+(a+\Delta a(t))cos(\theta ))\ddot{\theta }(t)&= -b\dot{\theta }^{3}(t)+c\theta (t) \\&\quad+(c_{\tau }+\Delta c_{\tau }(t)) \theta (t-\tau (t))+eu(t) \end{aligned}$$

The range of $\dot{\theta }(t)$ is assumed to satisfy $|\dot{\theta }(t)|<\varphi$, $\varphi =2$. *u*(*t*) is the control input. $\tau (t)=0.85+0.05 sin(10t)$ is the time-varying delay (thus, $\tau _{1}=0.8$, $\tau _{2}=0.9$, $d=0.5$). For the simulation purpose, the system parameter is given as $a=0.3$, $b=0.5$, $e=0.2$, $c=1$, $c_{\tau }=0.8$. As in Lin et al. (2006), we introduce new variables $x(t)=[x_{1}(t)\ x_{2}(t) \ x_{3}(t)]^{T}$ with $x_{1}(t)=\theta (t)$, $x_{2}(t)=\dot{\theta }(t)$ and $x_{3}(t)=\ddot{\theta }(t)$. The system is described by

$$\begin{aligned} \begin{bmatrix} 1&\quad 0&\quad 0 \\ 0&\quad 1&\quad 0 \\ 0&\quad 0&\quad 0 \end{bmatrix}{\dot{x}}(t) =\begin{bmatrix} 0&\quad 1&\quad 0 \\ 0&\quad 0&\quad 1 \\ c&\quad -bx_{2}^{2}(t)&\quad -1-acosx_{1}(t) \end{bmatrix}x(t) + \begin{bmatrix} 0&\quad 0&\quad 0 \\ 0&\quad 0&\quad 0 \\ c_{\tau }&\quad 0&\quad 0 \end{bmatrix}x(t-\tau (t)) + \begin{bmatrix} 0 \\ 0 \\ e \end{bmatrix}u(t) \end{aligned}$$

Then this system can be expressed exactly by the following fuzzy singular form with respect to uncertainties described by (2):

$$\begin{aligned} \left\{ \begin{aligned} &E{\dot{x}}(t)=\sum _{i=1}^{3}\mu _{i}(\xi (t))\{ (A_{i}+\Delta A_{i}(t))x(t)+(A_{\tau i}+\Delta A_{\tau i}(t))x(t-\tau (t))+B_{i}u(t)\} \\ &x(t)=\sum _{i=1}^{3}\mu _{i}(\xi (t))\phi _{i}(t) \end{aligned}\right. \end{aligned}$$

where

$$\begin{aligned} E&=\begin{bmatrix} 1&\quad 0&\quad 0 \\ 0&\quad 1&\quad 0 \\ 0&\quad 0&\quad 0 \end{bmatrix},\quad A_{1}=\begin{bmatrix} 0&\quad 1&\quad 0 \\ 0&\quad 0&\quad 1 \\ c&\quad -b(\varphi ^{2}+2)&\quad a-1 \end{bmatrix},\quad A_{3}=\begin{bmatrix} 0&\quad 1&\quad 0 \\ 0&\quad 0&\quad 1 \\ c&\quad 0&\quad a-1 \end{bmatrix} \\ A_{2}&=\begin{bmatrix} 0&\quad 1&\quad 0 \\ 0&\quad 0&\quad 1 \\ c&\quad 0&\quad -a-1-a\varphi ^{2} \end{bmatrix},\quad A_{\tau i}=\begin{bmatrix} 0&\quad 0&\quad 0 \\ 0&\quad 0&\quad 0 \\ c_{\tau }&\quad 0&\quad 0 \end{bmatrix},\quad B_{i}=\begin{bmatrix} 0 \\ 0 \\ e \end{bmatrix},\quad (i=1,2,3) \end{aligned}$$

The membership functions can be chosen as

$$\begin{aligned} \mu _{1}(t)=\frac{x_{2}^{2}(t)}{\varphi ^{2}+2}, \quad \mu _{2}(t)=\frac{1+cos(x_{1}(t))}{\varphi ^{2}+2}, \quad \mu _{3}(t) =\frac{\phi ^{2}-x_{2}^{2}(t)+1-cos(x_{1}(t))}{\varphi ^{2}+2} \end{aligned}$$

Here, we set $\rho =0.5$, $\lambda =1$ and assume that the parameters uncertainty matrices in $\Delta A_{i}(t)$ and $\Delta A_{\tau i}(t)$ in (2) are given as follows:

$$\begin{aligned} M_{i}&=\begin{bmatrix} 0.1&\quad 0&\quad 0 \\ 0&\quad 0.5&\quad 0 \\ 0&\quad 0&\quad 0.1 \end{bmatrix},\quad N_{11}=N_{13}=\begin{bmatrix} 0&\quad 0&\quad 0 \\ 0&\quad 0&\quad 0 \\ 0&\quad 0&\quad a \end{bmatrix} \\ N_{12}&=\begin{bmatrix} 0&\quad 0&\quad 0 \\ 0&\quad 0&\quad 0 \\ 0&\quad 0&\quad -a(\varphi ^{2}+1) \end{bmatrix},\quad N_{2i}=\begin{bmatrix} c_{\tau }&\quad 0&\quad 0 \\ 0&\quad 0.1&\quad 0 \\ 0&\quad 0&\quad 0.1 \end{bmatrix},\quad (i=1,2,3) \end{aligned}$$

In this example, considering the case of controller gain variation in the form of (33), the parameters are given as

$$\begin{aligned} M_{ai}=\begin{bmatrix} 0.1&\quad 0.1&\quad 0.2 \end{bmatrix}, \quad N_{ai}= \begin{bmatrix} 0.1&\quad 0.1&\quad 0.2 \\ 0.1&\quad 0.1&\quad 0.3 \\ 0.2&\quad 0.4&\quad 0.1 \end{bmatrix} (i=1,2,3) \end{aligned}$$

Then, according to Theorem 13 and by solving LMIs (35)–(43) with (44), we can obtain the feasible solution for Case I ($N=1$) as follows: (due to space consideration, we do not list all the matrices here)

$$\begin{aligned} X&=\begin{bmatrix} 0.2228&\quad -0.0943&\quad 0.0466 \\ -0.0943&\quad 0.1031&\quad -0.1530 \\ 0.0466&\quad -0.1530&\quad 1.4543 \\ \end{bmatrix},\quad \bar{P}_{1}=\begin{bmatrix} 0.2711&\quad -0.1386&\quad 0.2555 \\ -0.1386&\quad 0.1659&\quad -1.5745 \\ 0.2555&\quad -1.5745&\quad 23.7354 \\ \end{bmatrix} \\ \bar{Q}_{1}&=\begin{bmatrix} 0.0523&\quad -0.0247&\quad 0.0024 \\ -0.0247&\quad 0.0125&\quad -0.0083 \\ 0.0024&\quad -0.0083&\quad 4.8551 \\ \end{bmatrix},\quad \bar{S}_{1}=\begin{bmatrix} \ 0.0776&\quad -0.0373&\quad 0.0654 \\ \ -0.0373&\quad 0.0191&\quad 0.0032 \\ \ 0.0654&\quad 0.0032&\quad 3.3431 \\ \end{bmatrix} \\ \bar{S}_{2}&=\begin{bmatrix} 0.0715&\quad -0.0391&\quad -0.0026 \\ -0.0391&\quad 0.0361&\quad 0.0045 \\ -0.0026&\quad 0.0045&\quad 2.7343 \\ \end{bmatrix},\quad \bar{S}_{3}=\begin{bmatrix} 0.0731&\quad -0.0413&\quad -0.0013 \\ -0.0413&\quad 0.0433&\quad 0.0023 \\ -0.0013&\quad 0.0023&\quad 1.3438 \\ \end{bmatrix} \\ \bar{R}_{1}&=\begin{bmatrix} 0.6551&\quad -0.3024&\quad 0.2893 \\ -0.3024&\quad 0.2563&\quad -0.7339 \\ 0.2893&\quad -0.7339&\quad 3.5068 \\ \end{bmatrix},\quad \bar{R}_{2}=\begin{bmatrix} 0.5959&\quad -0.2666&\quad 0.2165 \\ -0.2666&\quad 0.2178&\quad -0.6041 \\ 0.2165&\quad -0.6041&\quad 2.9812 \\ \end{bmatrix} \\ V_{1}&=[-4.2929 \ \ \ \ \ 0.2507 \ -46.1158 ],\quad \varepsilon _{11}=0.3134, \varepsilon _{12}=0.3108,\varepsilon _{13}=0.3006 \\ V_{2}&=[-3.9275 \ -0.2329 \ -46.5635 ], \quad \varepsilon _{211}=1.5229, \varepsilon _{212}=0.2762,\varepsilon _{213}=1.5568 \\ V_{3}&=[-3.7714 \ -0.0943 \ -48.3501],\quad \varepsilon _{222}=2.3433, \varepsilon _{223}=1.5910,\varepsilon _{233}=1.5191 \\ \end{aligned}$$

Then, the feedback controller gains are designed as

$$\begin{aligned}&K_{1}=\begin{bmatrix}-58.2677&\quad -112.7529&\quad -41.7051 \end{bmatrix} \\&K_{2}=\begin{bmatrix} -59.5466&\quad -120.1667&\quad -42.7519 \end{bmatrix} \\&K_{3}=\begin{bmatrix}-58.2903&\quad -119.4432&\quad -43.9446 \end{bmatrix} \\ \end{aligned}$$

Similarly, according to Theorem 13 and by solving LMIs (35)–(43) with (45), we can obtain that the feedback controller gains in Case II are designed as:

$$\begin{aligned}&\bar{K}_{1}=\begin{bmatrix}-39.0312 \quad -64.8855 \quad -20.9107\end{bmatrix} \\&\bar{K}_{2}=\begin{bmatrix}-39.6921 \quad -69.1777 \quad -21.6299\end{bmatrix} \\&\bar{K}_{3}=\begin{bmatrix}-38.6646 \quad -68.7853 \quad -21.6868\end{bmatrix} \\ \end{aligned}$$

**Fig. 1:**
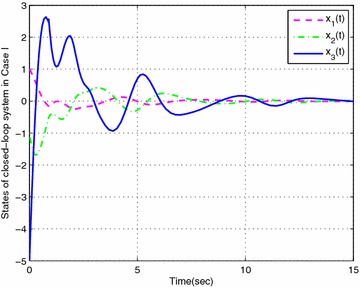
States response of closed-loop system with designed fuzzy controller in Case I

**Fig. 2:**
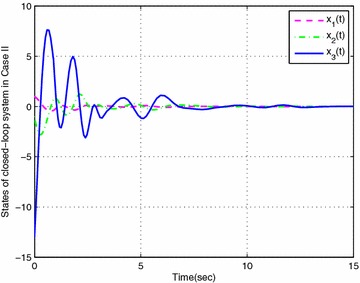
States response of closed-loop system with designed fuzzy controller in Case II

**Fig. 3:**
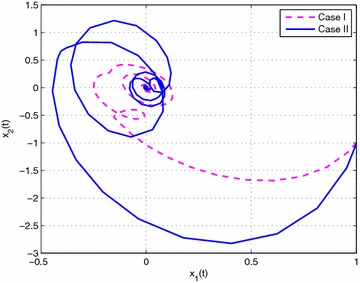
The phase portrait of closed-system states $x_{1}(t)$ and $x_{2}(t)$ in Case I and II

**Fig. 4:**
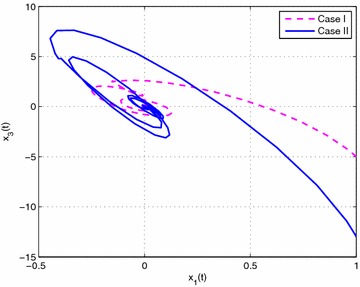
The phase portrait of closed-system states $x_{1}(t)$ and $x_{3}(t)$ in Case I and II

**Fig. 5:**
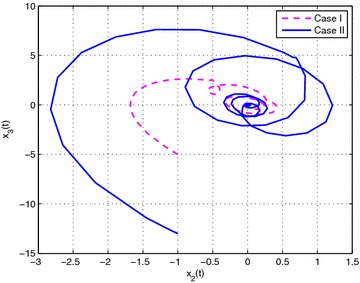
The phase portrait of closed-system states $x_{2}(t)$ and $x_{3}(t)$ in Cases I and II

Then, let the initial condition be $x_{1}(t)=1$, $x_{2}(t)=-1$, and the unknown matrix function $F(t)=F_{a}(t)=sin(t)$. The simulation results are given in Figs. 1, 2, 3, 4 and 5. Figures 1 and 2 plots the state trajectories of the closed-loop system with the obtained feedback gain matrices in Case I and Case II, respectively. The phase portraits of closed system are given in Figs. 3, 4 and 5. From the simulation result, it can be seen that the designed fuzzy controller not only makes the closed-loop system states converge to zero, but also effectively attenuate the uncertainty as expected.

## Conclusion

In this paper, the stability analysis and fuzzy stabilizing controller design for fuzzy singular systems with interval time-varying delay have been discussed. Based on improved delay partitioning method, new stability criteria for unforced fuzzy singular systems have been established. Then, the explicit expression of the desired fuzzy controller gains are also presented. All the obtained results reported in this paper are formulated in terms of strict LMIs, which can be readily solved using standard numerical software. Some numerical examples are provided to show the effectiveness of the proposed methods.
